# Whole blood transcriptome correlates with treatment response in nasopharyngeal carcinoma

**DOI:** 10.1186/1756-9966-31-76

**Published:** 2012-09-17

**Authors:** Adel M Zaatar, Chun Ren Lim, Chin Wei Bong, Michelle Mei Lin Lee, Jian Jiek Ooi, David Suria, Rakesh Raman, Samuel Chao, Hengxuan Yang, Soon Bin Neoh, Choong-Chin Liew

**Affiliations:** 1 Mount Miriam Cancer Hospital, 23, Jalan Bulan, Fettes Park, Tanjong Bungah, 11200, Penang, Malaysia; 2 Gleneagles Medical Centre, 1 Jalan Pangkor, Georgetown, 10050, Penang, Malaysia; 3 GeneNews Malaysia, 23, Jalan Bulan, Fettes Park, Tanjong Bungah, 11200, Penang, Malaysia; 4 GeneNews Limited, 2 East Beaver Creek Road, Building 2, Richmond Hill, Ontario, L4B 2N3, Canada; 5 Lam Wah Ee Hospital, Jalan Tan Sri Teh Ewe Lim, Georgetown, 11600, Penang, Malaysia; 6 Brigham and Women’s Hospital, Harvard Medical School, 75 Francis Street, Boston, USA

**Keywords:** Nasopharyngeal carcinoma, Microarray, Blood, Transcriptomics

## Abstract

**Background:**

Treatment protocols for nasopharyngeal carcinoma (NPC) developed in the past decade have significantly improved patient survival. In most NPC patients, however, the disease is diagnosed at late stages, and for some patients treatment response is less than optimal. This investigation has two aims: to identify a blood-based gene-expression signature that differentiates NPC from other medical conditions and from controls and to identify a biomarker signature that correlates with NPC treatment response.

**Methods:**

RNA was isolated from peripheral whole blood samples (2 x 10 ml) collected from NPC patients/controls (EDTA vacutainer). Gene expression patterns from 99 samples (66 NPC; 33 controls) were assessed using the Affymetrix array. We also collected expression data from 447 patients with other cancers (201 patients) and non-cancer conditions (246 patients). Multivariate logistic regression analysis was used to obtain biomarker signatures differentiating NPC samples from controls and other diseases. Differences were also analysed within a subset (n = 28) of a pre-intervention case cohort of patients whom we followed post-treatment.

**Results:**

A blood-based gene expression signature composed of three genes — LDLRAP1, PHF20, and LUC7L3 — is able to differentiate NPC from various other diseases and from unaffected controls with significant accuracy (area under the receiver operating characteristic curve of over 0·90). By subdividing our NPC cohort according to the degree of patient response to treatment we have been able to identify a blood gene signature that may be able to guide the selection of treatment.

**Conclusion:**

We have identified a blood-based gene signature that accurately distinguished NPC patients from controls and from patients with other diseases. The genes in the signature, LDLRAP1, PHF20, and LUC7L3, are known to be involved in carcinoma of the head and neck, tumour-associated antigens, and/or cellular signalling. We have also identified blood-based biomarkers that are (potentially) able to predict those patients who are more likely to respond to treatment for NPC. These findings have significant clinical implications for optimizing NPC therapy.

## Background

Nasopharyngeal carcinoma (NPC) is a squamous cell carcinoma arising in the nasopharyngeal epithelial lining, where the back of the nose meets the throat. The cancer is rare in most parts of the world, with an incidence of less than one per 100,000 populations in Europe and North America. In parts of Africa and in Asia, however, NPC is much more common. The highest incidence worldwide occurs in southeast China; in Hong Kong for example, NPC affects approximately 20–30 per 100,000 men and 15–20 per 100,000 women [1]. In Malaysia, NPC is the third most common cancer in men (after colon cancer and lung cancer), with an incidence of 15·9 per 100,000 in Chinese males in Malaysia [2].

The disease is more often diagnosed in men than in women, and tends to occur at an earlier age than do most cancers. In high-risk populations the risk of NPC increases slowly throughout the lifespan, with a peak incidence at 45–54 years. In moderate-risk groups, such as populations in North Africa, there is an additional peak in adolescence and youth (ages 10–20) [3].

NPC seems to involve a combination of etiological factors, both genetic and environmental [3,4]. The disease is strongly linked to Epstein-Barr virus (EBV), a herpesvirus transmitted by saliva and carried by 90% of the population. EBV is detected in plasma of 95% of patients with pre-malignant NPC lesions/tumour cells, and serology screening has been used for NPC screening in endemic areas [5,6].

The symptoms of NPC are non-specific, including neck mass, nasal and aural dysfunction and headaches, and clinical examination of the nasopharynx is difficult. Thus, more than 60% of patients with NPC present at locally advanced stages III and IV.

The awkward location of the nasopharynx also means that surgery is uncommon in NPC. Standard treatment of locoregional advanced NPC involves radiation therapy alone for earlier stages I and II cancer and radiation and concurrent cisplatin-based chemotherapy for later stages of the disease**.** Patients with stage I and II disease can usually be treated with radiotherapy alone, with excellent survival rates of 80–95% [7]. With chemoradiotherapy, patients with stage III and IV disease have reported 3-year survival rates of 70–80% [8,9]. In patients with recurrent or metastatic disease the prognosis is poor, with a median survival time of less than one year [7]. Undesirable complications of chemoradiotherapy for NPC can be severe and can limit patient compliance [8]. A blood test that could identify pre-symptomatic, earlier-stage NPC would help to increase patient survival and reduce treatment-related toxicity; a blood test that could predict patient response to therapy could increase compliance with treatment regimens.

In this report, we used blood samples to identify gene expression signatures for NPC and to predict patient response to treatment. Such a test would significantly improve the medical management of this disease.

## Methods

### Patients and blood samples

Blood samples were collected from patients with NPC recruited at Mount Miriam Cancer Hospital (Penang, Malaysia). Consent forms were obtained from all study participants according to protocols approved by the hospital’s Research Ethics Board.

We performed gene profiling and microarray analysis on 66 samples taken from patients with tumours confirmed as NPC by hospital pathologists, and for 33 controls (Table 1), collected between November 2006 and April 2010.

**Table 1:** Clinical characteristics of the patient cohorts for microarray hybridization

**Characteristics**	**NPC**	**Control**	P value*	**Control & 447 Other**	P value*
			**(NPC vs Control)**		**(NPC vs Control & 447 Other)**
No.	66	33		480	
Age - Median (Range)	51 (24–74)	31 (19–74)	<0·01	55 (19–86)	0.32
Malay	12 (18·2)	2 (6·1)	0·13	n/a	n/a
Chinese	45 (68·2)	30 (90·9)	0·01	n/a	n/a
Indonesian	8 (12·1)	0 (0·0)	0·05	n/a	n/a
Indian	0 (0·0)	1 (3·0)	0·33	n/a	n/a
Unknown	1 (1·5)	0 (0·0)	1·00	n/a	n/a
Male	49 (74·2)	20 (60·6)	0·17	242 (57.1)	0.01
Female	17 (25·8)	13 (39·4)	0·17	182 (42.9)	0.01
not available				56	

* P values for age and BMI were calculated using the Mann–Whitney test, P values for ethnicity, sex and medical conditions were calculated using Fisher’s exact test.

To obtain a gene signature specific to NPC, we included 447 expression profiles of samples with other conditions (27 bladder cancer; 10 breast cancer; 17 cervical cancer; 16 endometrical cancer; 40 ovarian cancer; 91 prostate cancer; 47 Crohn’s disease; 43 osteoarthritis; 38 rheumatoid arthritis; 85 cardiovascular disease; 20 schizophrenia; 13 miscellaneous other conditions).

### Blood collection, RNA isolation and RNA quality control

Peripheral whole blood (2x10 ml) was collected from patients in EDTA Vacutainer tubes (Becton Dickinson, New Jersey, USA), and RNA was isolated as described previously [10]. Isolated RNA was checked using 2100 Bioanalyzer RNA 6000 Nano Chip (Agilent Technologies, California, USA). Samples were excluded for subsequent microarray analysis that did not meet the following quality criteria: RIN > = 7·0; 28S:18S rRNA > =1·0. RNA quantity was determined by absorbance at 260 nm in a DU640 Spectrophotometer (Beckman-Coulter, California, USA).

### Microarray hybridization

Five micrograms of RNA from each sample were used for cDNA synthesis and hybridization, following the standard Affymetrix protocol (Affymetrix GeneChip, HG-U133 Plus 2·0). Hybridizations were assessed by the quality threshold for the Affymetrix GeneChip suggested by the manufacturer.

### Microarray analysis of NPC vs. controls and other diseases

Details of the statistical analysis are described in the Additional file 1.

### Microarray analysis of complete response to treatment (CR) vs partial response (PR) to treatment

Follow-up information from clinicians was available for 28 of the NPC cases. All but one of the patients had been treated with standard radiotherapy and 5–7 weeks cisplatin-based therapy (one patient received only radiotherapy), and the patients were followed for between one and three years. Clinical information for the cohort is presented in Table 2.

**Table 2:** Pathology information for the 28 samples

**Case**	**PR/CR**	**Tumour type**	**TNM Staging**
1	PR	Undifferentiated squamous cell carcinoma	T3NxMx
2	PR	Undifferentiated cell carcinoma WHO type III	T3N3Mx
3	PR	Moderately differentiated squamous cell carcinoma	T3N3Mx
4	PR	Undifferentiated Carcinoma	T3N3Mx
5	PR	Infiltrating, non-keratinising undifferentiated carcinoma; Loc adv NPC T1-2N2Mx with neck node mets, residual lesion	T3N3Mx
6	PR	Undifferentiated carcinoma ; CA nasopharynx stage III	T3N1Mx
7	PR	Moderately differentiated squamous cell carcinoma, keratinizing, NPC with Extensive right neck node mets; Residual disease and neck node; stable disease liver lesion	T2N3Mx
8	PR	Undifferentiated carcinoma WHO-3 , infiltrating	T2N1Mx
9	PR	Undifferentiated carcinoma WHO - 3, infiltrating; Loc adv NPC with neck node mets and multiple cranial nerces invol	T4N3Mx
10	PR	Undifferentiated carcinoma	T2N3Mx
11	PR	Poorly differentiated carcinoma	T2N?Mx
12	PR	Infiltrating, non-keratinizing undifferentiating carcinoma WHO type III tumour	T2N1Mx
13	PR	Poorly differentiated or anaplastic carcinoma	T2N1Mx
14	CR	Invasive, non-keratinising undifferentiated carcinoma WHO type III tumour	T3N2Mx
15	CR	Undifferentiated carcinoma, infiltrating; carcinoma of the nasopharynx, tumour involving the sphenoid bone & extending into the sphenoid sinus.	T4N2Mx
16	CR	Undifferentiated carcinoma	T2N2Mx
17	CR	Undifferentiated carcinoma, infiltrating, non-keratinizing WHO type III; Undiff NPC with retropharyngeal and left internal post jugular lymphadenopathy, for restaging.	T3N3Mx
18	CR	Undifferentiated carcinoma; Loc adv NPC T3N0Mx extending to the left parapharyngeal region	T3N0Mx
19	CR	Undifferentiated carcinoma	T4N1Mx
20	CR	Undifferentiated carcinoma	T3N1Mx
21	CR	Undifferentiated carcinoma (with left vocal cord palsy)	T3N2Mx
22	CR	Undifferentiated carcinoma (with cervical node metastases); NPC with neck node mets	T3N2Mx
23	CR	Undifferentiated carcinoma	T3N2Mx
24	CR	Non-keratinizing undifferentiated carcinoma, WHO type III, restaging	T2N1Mx
25	CR	Undifferentiated carcinoma; CT scan - neck node	T2N1Mx
26	CR	Undifferentiated carcinoma; Loc adv carcinoma of the nasopharynx with neck node mets, extension to parapharyngeal region right more than left and involvement of the ptergoid muscles	T3N2Mx
27	CR	Infiltrating non keratinising undifferentiated carcinoma; Tumour involv. Both FOR with parapharyngeal & rectopharyngeal extension	T3N1Mx
28	CR	Undifferentiated carcinoma; loc adv T4N1Mx. Tumour involv PNS, clivus, paratracheal & prevertebral muscles, ant nasal cavity and ext to both middle cranial fossa (extradural mass)	T4N1Mx

As evaluated with computed tomography scans taken at the last visit, 15 cases were classified as complete response to treatment (CR), that is, no evidence of disease was present, and 13 were classified as partial response to treatment (PR), that is, residual disease or metastasis was present. Gene profiles were analysed to identify a suite of biomarker genes capable of predicting a patient’s response to treatment. (Analysis is described in the Additional file 1.)

### Pathway analysis

Pathway analysis was performed using GeneSpring GX (version 10). BioPAX format pathways were imported into GeneSpring GX via http://biopax.org. The “Find Similar Pathway Tool” was used to identify pathways with considerable enrichment of the genes from our study. P-values were calculated using hypergeometric distribution or the Fisher’s exact test; the cut-off was set at < 0·05.

## Results

Of the 66 patients with NPC, there were more males than females (49 males, 17 females; see Table 1), a finding consistent with previous studies indicating that the incidence of NPC is higher in men than in women (male: female ratio = 3:1). We selected 66 samples for this study (36 newly diagnosed NPC (pre-treatment) and 30 post-treatment samples). Patient age, gender and other variables are shown in Table 1. To obtain genome-wide expression data for the samples, 66 hybridizations using Affymetrix GeneChip were performed.

### NPC gene signature identification

**Figure 1:**
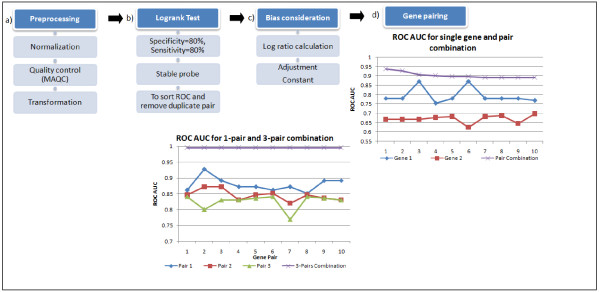
**Data Analysis Outline.** (**a**) Microarray gene profiling raw data were pre-processed for quality control before analysis. First, all samples were normalized using MAS5 algorithm and only probes flagged as “present” were retained. The “present” probes were then compared with the list generated in MAQC studies for Affymetrix Human U133 plus 2; non-overlapped probes were deemed unreliable and, therefore, excluded. Expression values of each probe were then logarithm transformed. (**b**) Logistic regression multivariate analysis of the gene expression values was performed to evaluate the AUC of each gene and of different multi-gene combinations. Significance of associations between gene expressions was determined using a logrank test. The best set of coefficient values that maximize the separation between the positive and negative groups were determined. Later, the log ratio calculation was determined in order to reduce the impact of possible noise (**c**). Thresholds were then set to evaluate sensitivity, specificity and the stability of the prediction. Two individual genes were combined to form a gene pair (**d**). Then the single pair of genes was coupled to form 2-pair and then 3-pair gene combinations. Logistic regression values were calculated for each gene pair, and we showed that in each case when genes were combined, the area under the curve (AUC ROC) increase.d

Microarray hybridizations were carried out to generate gene expression profiles for 66 blood samples from NPC patients, irrespective of treatment stage, and 33 control samples from Mount Miriam Cancer Hospital. Data analysis flow of the microarray data is shown in Figure 1 and in the Additional file 1. Using multivariate logistic regression analysis, we first selected 121 combinations of six probe sets with an AUC greater than 0·90 that separate NPC samples from unaffected controls and from patients with other diseases. The 121 combinations of six probe sets comprised 234 unique probe sets.

**Figure 2:**
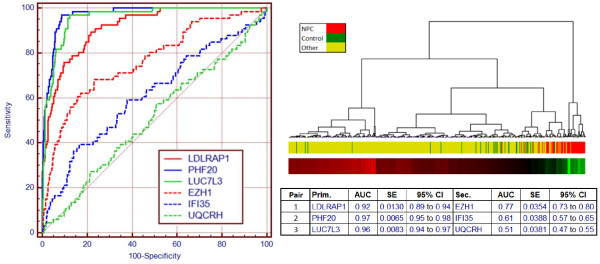
**ROCs of probes that contribute to differentiation of nasopharyngeal carcinoma from other conditions.** Combination of 6 genes with three genes appearing most frequently in all top-performing combinations LDLRAP1, PHF20 and LUC7L3. The additional three secondary genes have little NPC discrimination (ROC AUC: 0.51 – 0.77) but help suppress confounding factors. ROC AUC for each gene is listed in table. Dendrogram for the six-gene combination showing control samples dispersed throughout the “other” sample group with a separate cluster consisting mainly of NPC samples on the right. Heat map and clustering are based on results of 2-fold cross validation iterated 1000 times.

Of the 234 probe sets, we found that the three selected most frequently and in the best combinations mapped to genes LDLRAP1 (low density lipoprotein receptor adaptor protein 1), PHF20 (PHD finger protein 20) and LUC7L3 (cisplatin resistant-associated overexpressed protein, also known as CROP), with AUCs of 0·92, 0·97 and 0·96, respectively (Figure 2). The standard errors were relatively very small, at 0·013, 0·007 and 0·008, respectively. The cluster diagram in Figure 2 is based on a combination of these three primary genes with 3 secondary suppressor genes and shows that, to a large extent, the NPC samples stand apart from the controls, which are dispersed throughout the group of samples with other diseases.

This combination of three primary genes (LDLRAP1, PHF20, LUC7L3), together with their associated suppressor genes (EZH1, IFI35, UQCRH), was subjected to 2-fold cross-validation with 1000 iterations. The average ROC AUC was 0.98 (95% C.I. 0.98 – 0.99). An equivalent analysis using randomized NPC status achieved an average ROC AUC of 0.50 (95% C.I. 0.37 – 0.62). There was no overlap between these two distributions.

These 6 genes were run on qPCR for a subset of 26 controls and 44 NPC cases for which sufficient mRNA was available. The three gene pairs qPCR data confirmed the fold change directions observed in the microarray data with two of the three pairs remaining statistically significant (p-value < 0.01). The 3-pair combination maintained a high level of discrimination between cancer and controls with a 95% confidence interval (CI) for the ROC AUC of 0.75 to 0.93, overlapping that of the microarray data (95% CI: 0.91 to 1.00).

### Expression pattern difference reflecting treatment response

We subdivided a cohort of NPC patients prior to treatment (n = 28), according to the degree of patient response to treatment at one to three years of post-treatment follow-up. Analysis of this data identified gene pairs with ROC AUC ranging up to 0·94.

There were only 78 unique genes in the top 50-performing six-gene combinations, an enrichment factor of more than 3. This suggests that these genes are essential combination pairs and should have important biological roles in differentiating CR and PR. To elucidate such roles, we analyzed the 78 genes for their known involvement in relevant biological pathways. We found that three of the genes are involved in the 135-gene B-cell antigen receptor (BCR) pathway (p-val = 1.12E-04) and five genes are involved in the 176-gene epidermal growth factor receptor (EGFR) pathways (p-val = 0.024).

**Figure 3:**
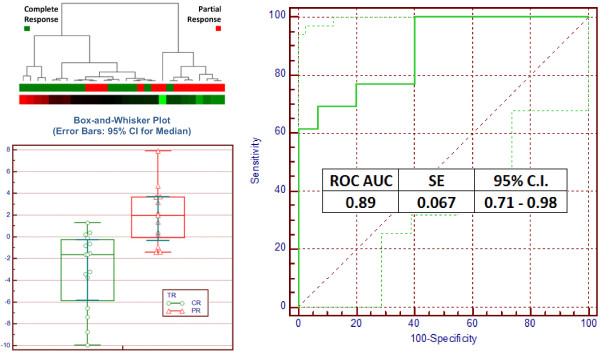
**Box-and-whisker median plot, hierarchical clustering results and ROC of Complete Response (CR) and Partial Response (PR) samples.** The box-and-whisker median (error bars: 95% CI for medians) plot for distribution of Complete Response (CR) and Partial Response (PR) for pair FOXP1 and STX16, showing good differentiation between the groups. The dendrogram represents the hierarchical clustering results of pre-intervention NPC samples. The coloured boxes directly below the dendrogram represent samples that show complete response (CR) to treatment and partial response (PR) to treatment, denoted in green and red, respectively. We found two major clusters in the samples; the cluster on the right consists of 8 of 13 (62%) PR samples (red) and the cluster on the left consists of 14 of 15 (93%) CR samples (green). Dot plot, heat map and clustering are based on results of 3-fold cross validation iterated 1000 times. The AUC for the single pair equation is 0·89, with a standard error of 0·067.

The four genes appearing most frequently in the combination were: forkhead box P1 (FOXP1, 34 combinations); egf-like module containing, mucin-like hormone receptor-like 2 (EMR2, 26 combinations); syntaxin 16 (STX16, 12 combinations); and N-acetylglucosamine-1-phosphate transferase (GNPTAB, 12 combinations). The best pair combination from these 4 genes with ROC AUC = 0.89, was FOXP1 and STX16 (Figure 3).

To reduce the risk of overfitting the data, we limited the remainder of the analysis to this single pair of genes.

We subjected the pair combination of FOXP1 and STX16 to cross validation analysis using 3-fold partitioning and iterated 1000 times. The average ROC AUC was maintained at 0.89 (95% C.I. range of 0.84 to 0.94). A “Null Set” analysis based on 1000 re-samplings of the data set with randomized labels produced a ROC AUC average of 0.50 (95% C.I. range of 0.29 to 0.71). There was a less than 1% overlap between the distribution of the cross validation using the actual data set and the “null set”.

## Discussion

The blood transcriptome is proving to be a valuable resource for biomarker identification and pharmacogenomics. In several studies we have shown that gene signatures obtained using blood mRNA can identify a variety of conditions including heart failure [11], cancer [10,12,13], inflammatory bowel disease [14,15], and psychiatric disorders [16-18]. In this study we have applied our methodology, using whole blood samples from NPC patients to compare gene expression patterns of NPC with unaffected controls and with other conditions and to compare blood gene expression patterns in NPC before and after radiotherapy and/or chemotherapy. Past research has identified tissue-based biomarkers for patient survival in NPC [19]. This will be the first study to develop a blood transcriptomic pharmacogenomic approach to guide treatment for NPC.

At the molecular level, LDLRAP1, PHF20 and LUC7L3 were the three probe sets most frequently selected for NPC discrimination. These genes have biological significance in NPC, as they are known to be involved in carcinomas of the head and neck, tumour-associated antigens, and/or cellular signalling. [20-26].

These results could throw light on biological pathways involved in patient response to NPC treatment. LUC7L3 [cisplatin resistant overexpressed protein (CROP)] is involved in RNA splicing or mRNA processing activities. Its expression is higher in cisplatin resistant cell-lines than in non-resistant cell-lines [23]. Cisplatin is believed to affect the sub-nuclear distribution of the protein, thereby interfering in RNA splicing and in the mRNA maturation process [24]**.** In this study, expression of LUC7L3 was found to be significantly lower in NPC samples than in controls and other cancer samples. Cisplatin is widely used to treat NPC patients. However primary and secondary cisplatin resistance is a major limitation to the use of this drug in cancer chemotherapy. Improved understanding of the mechanisms leading to cisplatin resistance may suggest molecular targets for therapeutic intervention and may facilitate prediction of response to therapy and individually tailored therapy [25].

Biological function analysis also indicates a significant enrichment of candidate genes involved in the BCR and EGFR1 pathways. The BCR pathway responds to specific antigens and is important for antibody production and immune responses [27]. Changes in expression of genes in this pathway may cause alterations in signal transmission within the cell, which can result in changes in B-cell production, cell growth and cell division. EBV, a herpesvirus strongly linked to NPC, replicates in B cells and epithelial cells and reportedly contributes to tumorigenesis [25]. Our finding of gene expression pathways in the BCR pathway in NPC could improve our understanding of NPC treatment responses, as changes in BCR pathways could lead to changes in EBV replication and tumour formation.

The results of this study have two major clinical implications. First, although the majority of newly diagnosed cases (75%) presented at late cancer stages, it is possible that the signature identified here may also indicate the presence of cancer at pre-symptomatic, earlier stages. This could lead to the development of a blood test for diagnosis at a stage at which the disease can be treated with less toxicity and higher chances of long-term patient survival.

The second important objective of the study was to determine whether expression information obtained from the blood of NPC patients can be used to further define appropriate treatment for individuals. In this context, the challenge was to detect any subtle differences evident in pre-intervention peripheral blood samples for NPC patients whose treatment response has been monitored for a period of time.

## Conclusion

In this study, NPC patients were diagnosed at later stage III and IV cancer (the stage at which most NPC patients are currently diagnosed). Whether the signature we have identified can also be detected in patients at an earlier, more treatable stage of the disease is an intriguing question for future research. A signature for early stage cancer could form the basis of a clinically useful blood test for the early diagnosis and screening of NPC.

Our blood-based gene expression signature also identifies those patients who are more likely to experience complete response to current radiation and chemotherapy regimens and those who can expect only a partial response to therapy. A test to identify complete responders could encourage patient compliance in the presence of treatment side-effects. Partial responders could be considered for assignment to new treatment plans or novel agents.

## Abbreviations

NPC, nasopharyngeal carcinoma; EBV, Epstein-Barr virus.

## Competing interests

Chun Ren Lim, Chin Wei Bong, Michelle Mei Lin Lee, Jian Jiek Ooi, David Suria, Samuel Chao, Hengxuan Yang and Choong-Chin Liew are all employed by GeneNews Limited, who sponsored this research. Choong-Chin Liew is Chief Scientist of GeneNews and also holds stock in the company. None of the other authors has any conflict of interest.

## Authors’ contributions

Adel M Zaatar: clinical concept, patient collection, data interpretation; Chun Ren Lim: analysis, literature search, writing; Chin Wei Bong: statistical analysis; Michelle Mei Lin Lee: experimental and technical support; Jian Jiek Ooi: technical support; David Suria: literature search, writing; Rakesh Raman: patient collection, clinical data interpretation; Samuel Chao: Statistical analysis; Hengxuan Yang: analysis, writing; Soon Bin Neoh: clinical interpretation; Choong-Chin Liew: study design, analysis, writing. All authors have read and approved the final manuscript.

## Supplementary Material

Additional file 1 Statistical analysis for NPC and treatment response discrimination. Click here for file
