# Supplementary material for: Whole blood transcriptome correlates with treatment response in nasopharyngeal carcinoma
Source: J Exp Clin Cancer Res. 2012 Sep 17;31(1):76. doi: 10.1186/1756-9966-31-76 (PMC3504566; doi:10.1186/1756-9966-31-76)
Supplement: Additional file 1 — Statistical analysis for NPC and treatment response discrimination. [file 1756-9966-31-76-S1.doc]

Additional file

Statistical analysis for NPC and treatment response discrimination

Microarray gene profiling preprocessing (Figure 1a). Normalization of samples was performed using MAS5 detection filter [1], which filtered out “absent” and “marginal” genes, retaining only “present” calls. Only those probe sets that are listed in the MAQC study [2] were retained, in order to ensure probe sets mapping to genes demonstrated to be replicable in qPCR are evaluated.

For the NPC discrimination experiment, we combined 447 “other disease” samples and 33 control samples. To balance the two non-NPC groups (controls and “other diseases”), we increased the weight of the smaller control groups by replicating the 33 control samples 12 times with added random Gaussian noise (SD = 0.1 in log2 space) to simulate uncertainty in the microarray system, producing the equivalent of 429 controls. NPC samples were not replicated.

Next, data transformation was conducted with base-two logarithm transformations. Finally, we performed a logistic regression (logreg) multivariate analysis of the expression values of the genes (Figure 1b).

Significance of associations between gene expressions was analyzed using logreg scores (Figure 1c). Candidate combinations were ranked according to the specificity corresponding to a fixed 80% sensitivity, the sensitivity for a fixed 80% specificity and the area under the receiver operating characteristic curve (ROC AUC). The regression coefficients in our analysis helped to adjust the relative proportion that each gene contributes to the logreg score. The method determined the set of coefficient values that best maximize the separation between the positive and negative groups. Combinations of genes were formed in a two-step process. First, genes were selected to form pairs. Then, combinations of pairs were evaluated for performance in discrimination.

For the treatment response discrimination experiment (CR vs. PR), candidate genes for pairing were selected as primary or secondary genes for a “primary-secondary” pair combination, following the “suppressor variable” approach [3,4].

Primary genes were selected from those that achieved the highest ROC AUC. Secondary genes were selected from those that achieved lower ROC AUC and had the highest root mean square (RMS) correlation to the set of primary genes. A set of approximately one hundred primary genes were selected based on the top ranked AUC (>0.70) genes. Excluding the primary genes, another set of approximately one hundred secondary genes were selected based on genes with lower AUC and highest RMS correlation values to the primary set. Similarly, combinations of pairs were formed using primary pairs and secondary pairs.

Since the possible number of combinations is extremely large, a Monte Carlo algorithm was used to evaluate candidates. Box-and-whisker plots, receiver operator characteristic plots and a heat map were made to graphically represent the results.

References

1 Stuart DP, Emma KS, Laura EE, Claire LW, Crispin JM: **The utility of MAS5 expression summary and detection call algorithms.** *BMC Bioinformatics* 2007, **8**:273.

2 MAQC Consortium: **The MicroArray Quality Control (MAQC) project shows inter- and intraplatform reproducibility of gene expression measurements.** *Nat Biotechnol* 2006, **24**:1151–1161.

3 Horst P: **The role of predictor variables which are independent of the criterion**. *Soc Sci Res Bull* 1941, **48**:431–436.

4 Friedman L, Wall M: **Graphical views of suppression and multicollinearity in multiple linear regression.** *Am Stat* 2005, **59**:127–136.
